# Alterations in Blood–Brain Barrier Integrity and Lateral Ventricle Differ in Rats Exposed to Space Radiation and Social Isolation

**DOI:** 10.3390/life14050636

**Published:** 2024-05-16

**Authors:** Austin M. Adkins, Zachary N. M. Luyo, Alayna J. Gibbs, Alea F. Boden, Riley S. Heerbrandt, Justin D. Gotthold, Richard A. Britten, Laurie L. Wellman, Larry D. Sanford

**Affiliations:** 1 Sleep Research Laboratory, Center for Integrative Neuroscience and Inflammatory Diseases, Pathology and Anatomy, Eastern Virginia Medical School, Norfolk, VA 23507, USA; adkinsam@evms.edu (A.M.A.); luyozn@evms.edu (Z.N.M.L.); bodenaf@evms.edu (A.F.B.); heerbrrs@evms.edu (R.S.H.); gotthojd@evms.edu (J.D.G.); wellmall@evms.edu (L.L.W.); 2 Pathology and Anatomy, Eastern Virginia Medical School, Norfolk, VA 23507, USA; gibbsaj@evms.edu; 3 Center for Integrative Neuroscience and Inflammatory Diseases, Radiation Oncology, Eastern Virginia Medical School, Norfolk, VA 23507, USA; brittera@evms.edu

**Keywords:** social isolation, space radiation, blood–brain barrier, lateral ventricle

## Abstract

The proposed Mars missions will expose astronauts to long durations of social isolation (SI) and space radiation (SR). These stressors have been shown to alter the brain’s macrostructure and microenvironment, including the blood–brain barrier (BBB). Breakdown of the BBB is linked to impaired executive functions and physical deficits, including sensorimotor and neurocognitive impairments. However, the precise mechanisms mediating these effects remain unknown. Additionally, the synergistic effects of combined exposure to SI and SR on the structural integrity of the BBB and brain remain unknown. We assessed the BBB integrity and morphology in the brains of male rats exposed to ground-based analogs of SI and SR. The rats exposed to SR had enlarged lateral ventricles and increased BBB damage associated with a loss of astrocytes and an increased number of leaky vessels. Many deficits observed in SR-treated animals were attenuated by dual exposure to SI (DFS). SI alone did not show BBB damage but did show differences in astrocyte morphology compared to the Controls. Thus, determining how single and combined inflight stressors modulate CNS structural integrity is crucial to fully understand the multiple pathways that could impact astronaut performance and health, including the alterations to the CNS structures and cell viability observed in this study.

## 1. Introduction

The future of space exploration and planned NASA operations, such as the proposed Mars missions, will require crew members to log longer mission times and travel deeper into space than ever before. As such, crews will also experience increased exposure to multiple inflight stressors, including social isolation (SI) and space radiation (SR). Moreover, these missions will likely offer new challenges and obstacles that NASA and crews have not yet encountered [1].

SI and SR have been reported to alter immune system functionality. SI alone induces neuroinflammation and microglial overactivation [2]. SI can increase redox stress and levels of pro-inflammatory cytokines (e.g., TNF-α) [3]. Exposure to SR can induce neuroinflammation by elevating microglial activation [4]. Increases in neuroinflammation related to SI and SR can induce changes to the brain’s macrostructure and microenvironment [5,6,7]. Significant alterations to CNS structural integrity have been associated with exposure to either SI and SR, including changes in tissue microenvironments [8] and regional morphology [9,10].

Individually, SI [11] and SR [8,12] have been reported to disrupt the blood–brain barrier (BBB). The BBB is an assembly of endothelial cells, blood vessels, astrocytes, and pericytes, which provides a semi-permeable border to regulate the movement of particles and cells into the CNS [13], thereby maintaining CNS homeostasis [14]. In contrast, the breakdown of the BBB leads to cognitive dysfunction and increases in neuroinflammation [15] and is associated with multiple neuropsychiatric disorders [16]. However, the precise mechanisms mediating these effects remain unknown. Therefore, alterations to immune functionality by exposure to SI and SR could reduce an astronaut’s ability to maintain adequate levels of performance during missions that could not only affect crew health but mission success through several pathways.

Ultimately, SI and SR-induced immune system changes will likely result in significant alterations to the brain’s structural integrity, which could lead to increases in anxiety, issues with memory processing, and impairments of cognitive and executive functions. However, the potential for SI and SR interactions to change immune system responses remains unknown. Given the dynamic environment of space missions, multiple stressors could have synergistic effects that interact in unknown ways that may result in differential outcomes. Thus, determining how single and compound inflight stressors interact to modulate the immune system and its relationship to structural alterations in the brain is crucial to fully understand the pathways impacting astronaut performance and health. In this experimental study, we investigated how SI and SR, and their combination, modified immune system function and its relevance to BBB integrity and brain homeostasis. Our goal was to assess how spaceflight stressors impact the BBB in ways that could alter brain function in astronauts.

## 2. Materials and Methods

### 2.1. Subjects

Male, outbred, Wistar strain rats (8–9 months old at the time of study, n = 4–5 per group) obtained from Hilltop Lab Animals, Inc. (Scottdale, PA, USA) were used in this study [17]. The rats were either subjected to SI (visual barriers between cages) or individually housed (as a Control group). SI began at least eight weeks prior to experimentation and was maintained throughout the study as previously described [17]. Separate groups of rats received a single dose of SR (15 cGy simplified 5-ion galactic cosmic radiation (GCRsim), Brookhaven National Laboratory (BNL); Long Island, NY) and were either individually housed or subjected to SI (dual flight stressors (DFS)). Other than irradiation, all the experimental manipulations and measures were conducted at Eastern Virginia Medical School in 2021–2022. Food and water were available ad libitum. The housing rooms were kept on a 12:12 light:dark cycle and the ambient temperature was maintained at 24.5 ± 0.5 °C. All the procedures were conducted in accordance with the National Institutes of Health Guide for the Care and Use of Experimental Animals and were approved by Eastern Virginia Medical School’s Institutional Animal Care and Use Committee (Protocol#: 19-018).

### 2.2. Euthanasia

The rats were euthanized via isoflurane sedation (inhalant: 5%, ≤5 min duration) and cardiac perfusion with 1X PBS. Their brains were extracted and halved along the longitudinal fissure. The right hemisphere was used for analyses not discussed in this study. The left hemisphere was prepared as described in detail below.

### 2.3. Histology

The left hemisphere of the brain was fixed in 10% formalin solution at 4 °C for 24 h and then processed and paraffin-embedded for the subsequent histological analysis (n = 4–5 in each group). Five μm sagittal tissue sections through the limbic system were obtained via microtomy and mounted on glass slides (two sections per slide) for confocal or light microscopy.

### 2.4. Confocal Microscopy

Quadruple-label immunofluorescence staining was used to visually assess the potential effects of SI and SR on BBB integrity. The slides were first run through a deparaffinization/hydration sequence as follows: xylene 2 × 5 min each, 100% EtOH 2 × 2 min each, 95% EtOH 2 × 1 min each, 70% EtOH 2 × 1 min each, and dH_2_O 2 × 1 min each. The slides were then quenched twice to reduce the fixative-induced auto-fluorescence by eliminating the free aldehyde groups using a 1% solution of NaBH_4_ in 70% EtOH for 30 min at room temperature (RT) followed by a solution containing 0.375% glycine and 0.267% NH_4_Cl in dH_2_O for 10 min. Next, the tissue sections were soaked in the UltraCruz^®^ blocking reagent for 30 min to eliminate potential background staining due to non-specific antibody binding. In between each solution, the tissue sections were quickly rinsed 3× each in 1X PBS. The tissue sections were then stained at RT for 1 h each with the following optimized concentrated antibodies: Fibrinogen (Diluted 1:600 in PBS. Biorbyt; Cat. #orb4255), Glut-1 (Diluted 1:200 in PBS. abcam; Cat. #ab195020), and GFAP (Diluted 1:400 in PBS. Novus Biologicals; Cat. #NBP2-34401AF488). In between each antibody incubation, the tissue sections were quickly rinsed 3× each in 1X PBS followed by a 5 min incubation in the glycine-containing quenching solution above. Following the final incubation, the tissue sections were covered in a Fluoroshield™ mounting medium containing DAPI (Abcam; Cat. #ab104139) and incubated for 5 min at RT and then cover-slipped and stored at 4 °C until imaged via confocal microscopy.

Stained tissue sections were imaged using a Zeiss Axio Observer Z.1 confocal microscope and ZEN Black acquisition software (version 2.1 SP3, Carl Zeiss, Inc.; Dublin, CA, USA) at 40× magnification. Eight 1024 × 1024 images including the basolateral amygdala, caudate putamen, hippocampus, and medial prefrontal cortex per treatment group were obtained. The acquired images were then uploaded to the ZEN Blue analysis software (version 3.7, Carl Zeiss, Inc.; Dublin, CA, USA) and the following parameters were assessed: total number of vessels, total number of astrocytes, total amount of fibrinogen extravasation, vesicular diameter, and astrocyte morphology (number of projections, distance of projections, and number of branches from projections).

The total number of vessels and astrocytes within each image were calculated using the cell counting tool within ImageJ (Version 2.14.0/1.54f) by manually “marking” each cell with the counter function. The counts were automatically tallied through ImageJ. The vesicular diameter was automatically calculated using the ImageJ measuring where a line was drawn across the width of the vessel. The number of astrocyte projections and branches within each image were calculated using the cell counting tool within ImageJ by manually “marking” each projection or branch with the counter function. Astrocyte projections were considered to be any protrusion that was directly connected to the cell body. Astrocyte branches were considered to be any outgrowth from a projection. The distance of each astrocyte projection was automatically calculated using the measuring tool within ImageJ, which was used to draw a line from the base of the astrocyte cell body to the furthest point of the projection.

### 2.5. Light Microscopy

H&E staining was used to assess the morphological changes in the brain. Following a deparaffinization/hydration sequence consisting of 60 dips in xylene, 20 dips in 100% EtOH, 10 dips 100% EtOH, 10 dips in 95% EtOH, 10 dips in 70% EtOH, and 10 dips in dH_2_O, the slides were submerged for 2 min in filtered modified Harris hematoxylin. The slides were then briefly rinsed with tap water and dipped 10× each in bluing reagent followed by dH_2_O. The slides were again briefly rinsed with tap water and then dipped 20× in eosin. Following a final dehydration sequence consisting of 10 dips in 95% EtOH, 20 dips in 100% EtOH, and 30 dips in xylene, the slides were cover-slipped.

The H&E-stained sagittal tissue sections (Lateral 1.4 mm, Bregma −0.45 mm) were imaged on a Nikon Eclipse E800 using the RT Slider SPOT Camera and SPOT acquisition software (version 3.1, Spot Imaging; Sterling Heights, MI, USA) light microscope at 2× magnification. Six 120 × 120 images per treatment group of the LV were obtained. All the images were then uploaded to ImageJ (Version 2.14.0/1.54f) software for analysis of the following parameters: LV total area, perimeter, width, and height, as well as number of endothelial cells.

The total area and perimeter of the LV for each image were automatically calculated using the ImageJ “freehand” selection tool to draw the area around the LV. The measuring tool was then used to draw a line within the area of the LV to automatically calculate the width and height for each image. The number of endothelial cells within the LV for each image were calculated using the cell counting tool within ImageJ by manually “marking” each cell with the counter function. The counts were automatically tallied through ImageJ.

### 2.6. Statistical Analyses

The data were analyzed with a one-way ANOVA with the treatment (Control, SI, SR, and DFS) as between factors. Tukey’s post hoc multiple comparisons test was performed when indicated by a significant ANOVA. All the ANOVAs were generated using GraphPad PRISM software (Version 9.4.1).

## 3. Results

### 3.1. Blood–Brain Barrier (BBB) Integrity

**Figure 1:**
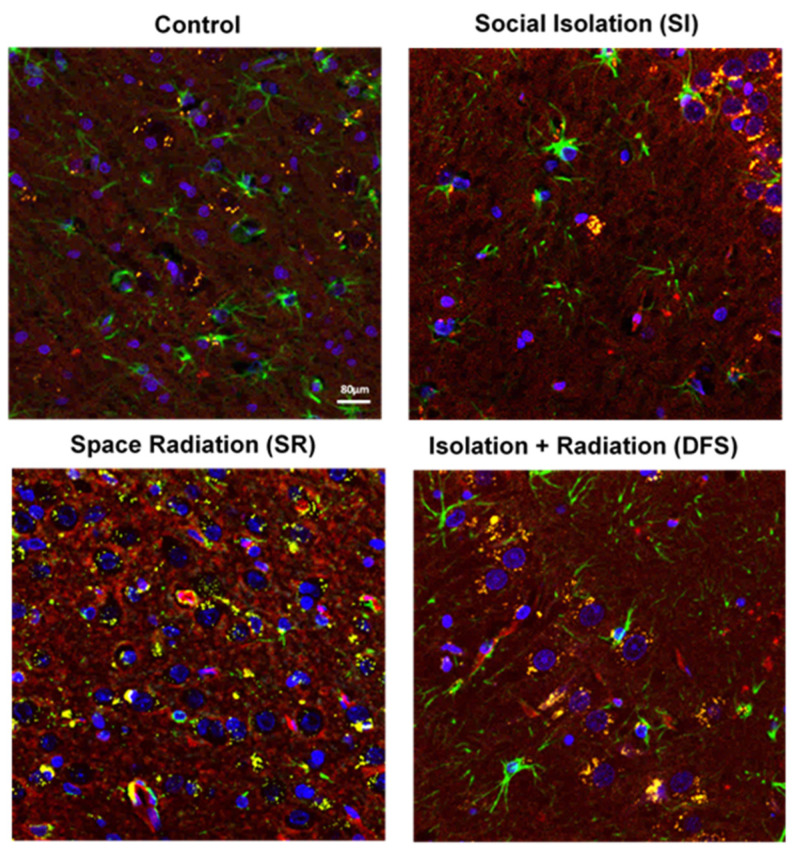
**SR-induced BBB damage and astrocyte death was ameliorated by SI.** Images of representative tissue slices within the limbic area of the brain showing quadruple-label immunofluorescence stained with GFAP (green), fibrinogen (yellow), Glut-1 (red), and DAPI (blue) displaying differences in vascular permeability in each treatment group. All images are at 40× magnification. Scale bar = 80 µm.

**Figure 2:**
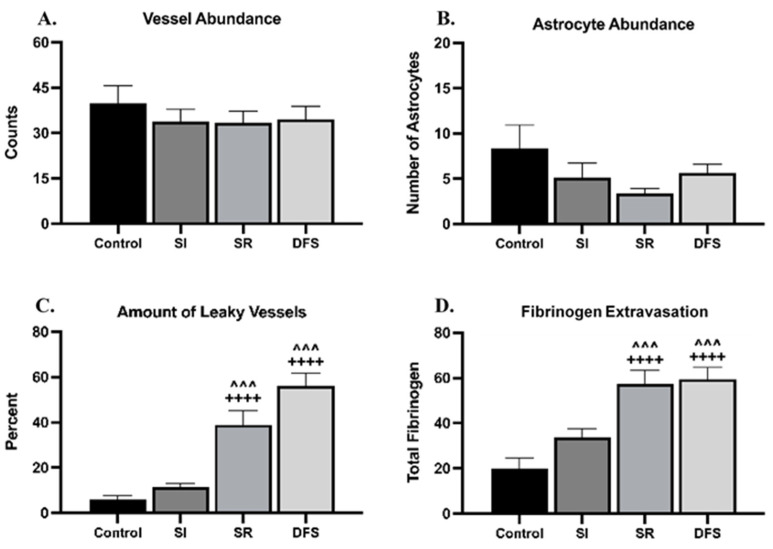
**Irradiated animals had increased leaky vessels and fibrinogen extravasation in the brain.** Graphs plotting the relative (**A**) vessel abundance (counts) ± SEM, (**B**) astrocyte abundance ± SEM, (**C**) % leaky vessels ± SEM, and (**D**) fibrinogen extravasation ± SEM based on quantified immunofluorescence staining amounts in the brain in each treatment group. Significant differences compared to Control: ++++ *p* < 0.0001. Significant differences compared to SI: ^^^ *p* < 0.001.

Figure 1 provides representative images for each treatment group stained to examine the potential damage to the BBB. The rats exposed to SR (15cGy) had a reduction in astrocytes compared to the Control group (indicated by low GFAP staining (in green)). Astrocyte loss was also associated with increased fibrinogen extravasation (indicated in yellow) outside of the vasculature into the brain parenchyma. Interestingly, there was a rescue of astrocytes in the DFS animals, but this was still associated with increased fibrinogen extravasation. There was no apparent astrocyte loss or increase in fibrinogen extravasation in the SI group compared to the Controls. The Control animals did not exhibit any evident insults to the BBB. The quantification of these observations revealed no difference in the total vessel abundance (*p* = 0.41) (Figure 2A) or number of astrocytes (*p* = 0.201) (Figure 2B). However, the ANOVA revealed significant differences of treatment in the percent of leaky vessels (indicated by positive fibrinogen staining (in yellow) outside of the vessel) (F_3,28_ = 29.49; *p* < 0.0001). Tukey’s post hoc test revealed that the SR and DFS groups had an increased percentage of leaky vessels compared to the Control and SI groups (*p* < 0.0001 and *p* < 0.001, respectively) (Figure 2C). The percentage of leaky vessels was also positively correlated with increased fibrinogen extravasation. The ANOVA revealed significant differences for the treatment group in the total amount of fibrinogen leaking from the vessels (F_3,28_ = 15.17; *p* < 0.0001). Tukey’s post hoc tests revealed that the SR and DFS groups had increased fibrinogen extravasation compared to the Control and SI groups (*p* < 0.0001 and *p* < 0.001, respectively) (Figure 2D). Additional images showing separated color channels can be viewed in Supplementary Figure S1.

### 3.2. Astrocyte Morphology

**Figure 3:**
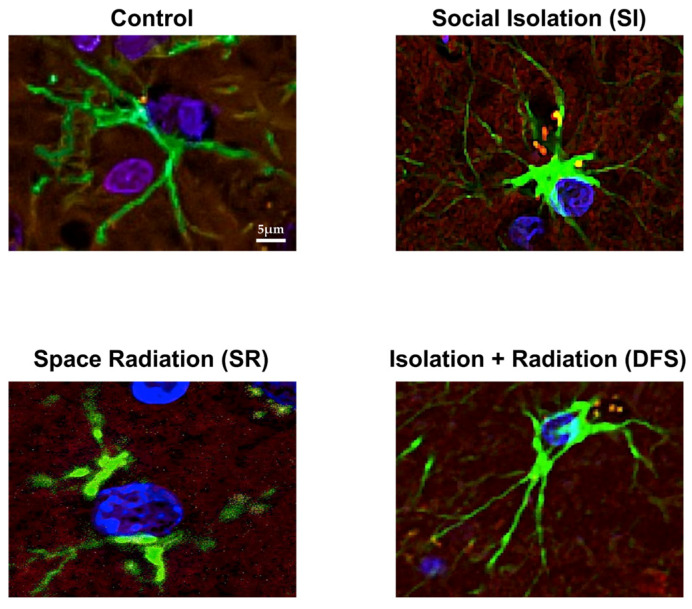
**SI and SR differentially altered astrocyte morphology.** Immunofluorescent images of individual astrocytes stained with GFAP (green) displaying differences in astrocyte morphology in each treatment group. Additional background staining includes fibrinogen (yellow), Glut-1 (red), and DAPI (blue). All images were acquired at 40× magnification and zoomed to focus on a single astrocyte. Scale bar = 5 µm.

**Figure 4:**
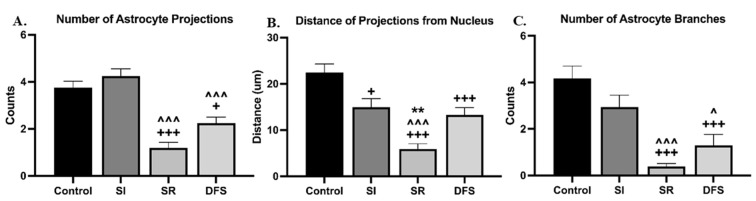
**The average number of projections and branches from astrocytes differed between SI and SR exposure.** Graphs plotting the (**A**) relative number (counts) of projections from an astrocyte soma ± SEM, (**B**) average distance from the soma of each projection ± SEM, and (**C**) relative number (counts) of branches off each projection ± SEM based on the quantified immunofluorescence staining amounts in each treatment group. Significant differences compared to Control: + *p* < 0.05, +++ *p* < 0.001. Significant differences compared to SI: ^ *p* < 0.05, ^^^ *p* < 0.001. Significant differences compared to DFS: ** *p* < 0.01.

Further investigation into the astrocytes between the groups also revealed morphological differences. The Control animals appeared to have a protoplasmic-like morphology with long, multi-branched radial projections [18,19]. The SI and DFS animals had a more fibrous morphology with an enlarged cell body and unilateral projections that were shorter, thinner, and less branched that showed increased GFAP staining intensity [18,19]. The astrocytes in the animals exposed to SR alone exhibited increased damage, including a dramatic loss of projections, or death (Figure 3). The quantification of these results via ANOVA revealed significant differences in the treatment group (F_3,78_ = 27.65; *p* < 0.0001). Tukey’s post hoc comparisons revealed astrocytes in the SR (*p* < 0.0001 compared to Control and SI) and DFS (*p* = 0.01 compared to Control and *p* < 0.001 compared to SI) groups had fewer projections (Figure 4A).

The analyses of the projection length via the ANOVA revealed significant differences for the treatment group (*F*_3,95_ = 19.88; *p* < 0.0001). Tukey’s post hoc tests revealed that the SI (*p* = 0.02), SR (*p* < 0.0001), and DFS (*p* < 0.001) groups had significantly shorter projections than the Control animals and fewer branches compared to the Control (*p* < 0.001) and SI (*p* < 0.001) groups, while the SR animals also had significantly shorter projections compared to the SI (*p* < 0.0001) and DFS (*p* = 0.001) groups (Figure 4B). Furthermore, the ANOVA analyses of the astrocyte branching revealed significant differences for the treatment group (*F*_3,78_ = 22.56; *p* < 0.0001). Tukey’s post hoc tests revealed that compared to the Control and SI groups, the SR (*p* < 0.0001) and DFS (*p* = 0.01) groups had significantly fewer branches (Figure 4C). A summary of these results is provided in Supplementary Table S1.

### 3.3. Brain Morphology

**Figure 5:**
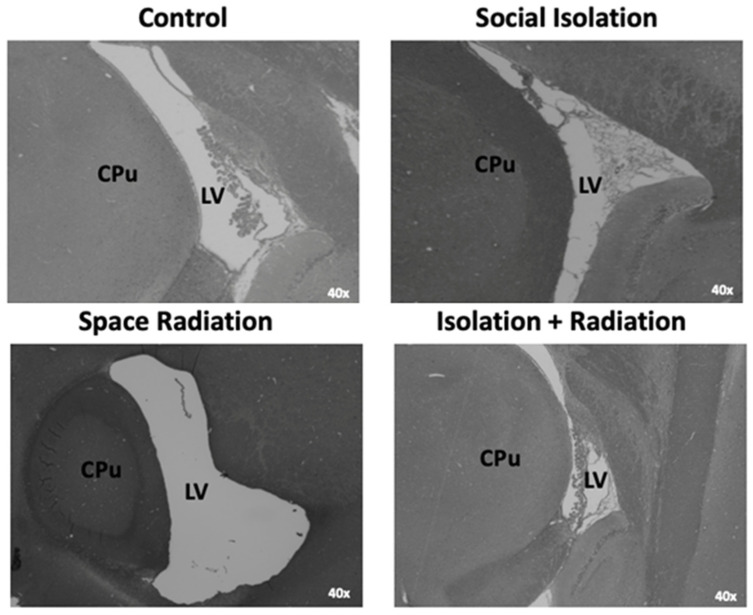
**SR exposure resulted in an enlarged lateral ventricle (LV), which was ameliorated by a combined exposure to SI.** Light microscopy images of sagittal brain sections (Lateral 1.4 mm, Bregma −0.45 mm) stained with H&E displaying morphological differences in LV area in each treatment group. Arrowheads point to endothelial cells within LV. CPu—caudate putamen. All images are at 2× magnification. Scale bar = 1 mm.

**Figure 6:**
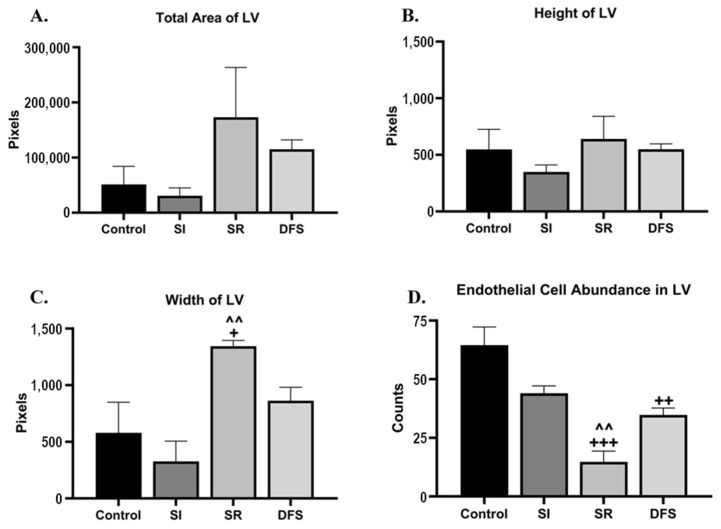
**SR animals had an increased lateral ventricle (LV) size that was rescued by DFS.** Graphs plotting the (**A**) average total area of LV ± SEM, (**B**) average height of LV ± SEM, (**C**) average width of LV ± SEM, and (**D**) average number of endothelial cells within LV ± SEM based on quantified H&E staining amounts in the brain for each treatment group. Significant differences compared to Control: + *p* < 0.05, ++ *p*<0.01, and +++ *p* < 0.001. Area measures are indicated in Pixels. Significant differences compared to SI: ^^ *p* < 0.01.

When investigating the morphological differences between the treatment groups, we found that the SR animals had a 1.5- to 5.6-fold enlargement of the lateral ventricle (LV) compared to any other group (Figure 5). Interestingly, we found that these deficits were ameliorated when SR was combined with SI (DFS), similar to the findings for astrocytes discussed above. The quantification of these results analyzed by ANOVA did not reveal significant differences in the total area of the LV for any group (*F*_3,12_ = 1.718; *p* = 0.2164) (Figure 6A). However, further investigation into the directionality of the LV enlargement revealed significant differences in the treatment group (*F*_3,12_ = 8.116; *p* < 0.01). Tukey’s post hoc tests that revealed significant differences in the LV size between the SR group compared to the other groups was in the medio-lateral direction (*p* < 0.05 compared to Control and *p* < 0.01 compared to SI, respectively) but not in the dorso-ventral direction (*p* = 0.53) (Figure 6B and Figure 6C, respectively).

We also found that the SR animals had a 2.4- to 4.4-fold reduction in endothelial cells lining the LV compared to any other group. These deficits were also ameliorated when SR was combined with SI (DFS), similar to the findings for the astrocytes discussed above. The ANOVA analyses of the quantification of the number of endothelial cells within the LV revealed significant differences for the treatment group (F_3,12_ = 17.15; *p* = 0.0001). Tukey’s post hoc tests revealed that the SR animals had significantly fewer endothelial cells in the LV compared to the Control (*p* < 0.0001) and SI (*p* < 0.01) animals. The DFS animals also had significantly fewer endothelial cells in the LV compared to the Control animals (*p* < 0.01) (Figure 6D).

## 4. Discussion

The present study provides comparative data for the effects of SI, SR, and DFS relative to the Control on measures of BBB integrity and on astrocyte and gross brain morphology in rats. The Control and SI rats did not differ in astrocyte numbers or show differences in fibrinogen extravasation or in gross brain morphology but did differ in astrocyte morphology. The astrocytes in the Control animals had protoplasmic-like morphology with long, multi-branched radial projections [18,19], whereas the astrocytes in both the SI and DFS (which also experienced SI) animals had a fibrous morphology with an enlarged cell body and unilateral projections that were shorter, thinner, and less branched with increased GFAP staining intensity [18,19]. By comparison, the SR and DFS rats exhibited both an increased percentage of leaky vessels and fibrinogen extravasation relative to the Control and SI rats, and the astrocytes in the rats exposed to SR alone exhibited greater astrocyte damage, including a loss of projections and death. The SR rats also had an enlarged LV in the medial-to-lateral direction and both the SR and DFS rats had a reduction in endothelial cells lining the LV compared to the Control, and in SR rats was also reduced compared to SI rats. Together, these findings suggest that the greater loss of BBB integrity and structural changes in the brain were associated with greater astrocyte damage and loss.

Overall, these data indicate that SR exposure had a negative impact on the integrity of brain macro- and microstructures. The altered morphology induced by SR appeared to disrupt both the vascular and lymphatic systems, observed by increased BBB permeability and LV enlargement, and was associated with a loss of supporting cells, including astrocytes and endothelial cells. Interestingly, some of the deficits observed in the SR group were marginally rescued in the DFS group. We have also observed a similar effect in some behavioral tasks [17], though the mechanisms are not yet known. Despite this partial rescue, compared to the Control and SI groups, the DFS animals still had a significant reduction in structural integrity. The differences in the BBB morphology exhibited in the group that was exposed to SI alone were mainly related to altered astrocyte morphology compared to the Control group.

The breakdown of the BBB has been associated with cognitive dysfunction and increases in neuroinflammation [15]. Studies have shown the negative effects of singular SR [20,21] and SI [11,22,23] exposure on BBB integrity. Specifically, SR has been shown to target the microvascular system and disrupt endothelial barrier function by uncoupling important cell adhesion molecules (e.g., PECAM-1 and CD31) [8]. SI has been reported to increase levels of pro-inflammatory cytokines [22] and reactive oxygen species (ROS) [23] in the brain.

Our study found similar evidence for the disruption of the microvasculature system by SR. However, we also found that BBB permeability in this group was heavily associated with astrocyte viability and/or altered functionality (decrease in, or complete loss of, projections) in astrocytes that survived. These SR-induced changes were heavily associated with increased vessel leakage and fibrinogen relocation into the brain parenchyma. Other studies have reported that SR can induce astrocyte senescence [24]. In our study, it is possible that the SR-induced loss and/or senescence of astrocytes dysregulated the vascular endothelium, which led to increased BBB permeability. Interestingly, dual exposure to SI and SR (DFS) ameliorated the astrocyte loss observed in SR alone but was associated with an altered, fibrous-like astrocyte morphology and increased vessel leakage. The fibrous morphology was also found in the animals that experienced SI alone, but this was not coupled with fibrinogen extravasation. To our knowledge, this study is the first to report chronic individual or combination effects of SI and SR on astrocyte viability and morphology in association with BBB regulation in vivo [25].

Astrocytes are integral in maintaining CNS homeostasis by providing structural support, supplying energy and metabolites, participating in immune responses [26], and regulating vasculature endothelial responses [25,27]. The existence of two basic subtypes of astrocytes in rodents has been established [18]. Protoplasmic astrocytes possess highly branched projections that can extend to enwrap synapses, as well as blood vessels, to form the outermost layer of the BBB. This allows for the regulation of both synaptic functions and the regulation of blood flow/endothelium integrity. Fibrous astrocytes possess unilateral, thin projections that are less branched. While the function of this subtype is still not clear, its projections are mainly associated with blood vessel interactions similar to the protoplasmic subtype. However, they create fewer connections with vessels compared to the protoplasmic subtype [28] and are associated with increased GFAP staining [18,19]. This evidence could explain the increased BBB permeability that still occurred in the SI and DFS groups compared to the Control group. Previous studies have shown that SI alone can cause astrocytes to become hyperactive, and this change in astrocyte function by SI has been linked to suppressed memory formation [29]. In our previously published work [17], SI animals experienced blunted learning in sensorimotor tasks, though this was not a chronic impairment. Further investigation into these alterations is required to fully understand the effects of SI on astrocytes.

We also found that SR alone induced LV enlargement, which was also associated with a dramatic loss of endothelial cells within the LV. Unlike in the BBB, these deficits appeared to be rescued by DFS exposure. Previous studies have reported that prolonged spaceflight altered cerebrospinal fluid (CSF), specifically through an enlargement of the LV and decreased area of subarachnoid spaces [30]. The negative effects of SR on the endothelial barrier and vascular integrity have also been previously reported [31,32]. To our knowledge, this study is the first to report combination effects of SI and SR (DFS) on endothelial cell viability and LV morphology.

BBB dysfunction has been linked to the pathogenesis of multiple neuropsychiatric disorders [16,33,34] and to more severe anxiety symptoms [16,34]. Indeed, the SR and DFS animals in this study exhibited increased BBB damage as well as increased anxiety across multiple behavioral paradigms [17,35]. Elevated anxiety has been previously reported in animals exposed to GCR [4,36]. Anxiety-related disorders and symptoms have also been associated with endothelial dysfunction in multiple organ systems (e.g., in the LV) [37,38]. Additionally, altered CSF flow and brain lymphatics have been linked to multiple neurodegenerative and neuromuscular diseases [39,40]. Mechanistically, this may be through a loss of tight junction proteins, as previous studies have shown that a loss of these proteins (e.g., Cldn5) induced anxiety-like behaviors [41].

Alterations in immune system functionality could also be associated with these morphological changes as previously discussed. Immune system activation has been shown to negatively impact mood and behavior by increasing anxiety [42,43,44], fatigue, and emotional dysregulation [45]. Given that executive functions play an important role in regulating behavior, motivation, impulses, and arousal, astronauts with alterations in the BBB and LV may exhibit reduced motivation and possibly aberrant or impulsive behavior. However, further investigation into these mechanisms is necessary to understand fully how BBB and LV dysfunction leads to increased altered behaviors.

Interestingly, the reported effects of SI alone on tight junction proteins have varied across studies. Alshammari et al. [11] reported that SI increased Cldn5 and tight junction proteins in the hippocampus, which could result in a tighter BBB. However, other work has reported that SI beginning on post-natal day 21 decreased Cldn5 and increased BBB breakdown and microglial activation in the amygdala relative to group-housed female mice [46]. Other studies have reported similar decreases in BBB structural components [47] produced by SI, which would be consistent with greater deficits in combined stressor rats. Our results are consistent with these later findings, though we did not find additional significant increases in leaky vessels and fibrinogen extravasation in DFS rats.

Our study has limitations. We conducted analyses on a relatively small number of animals per condition. Thus, there is the possibility that we missed some alterations that could have achieved significance with larger sample sizes. However, even with a smaller n, we observed significant alterations in the BBB parameters in the SR and DFS rats. Our study also was primarily descriptive and did not assess the functional significance of the alterations that we observed. The changes we observed predict likely impairments in BBB function; however, additional studies will be needed to assess and quantify the extent of functional impairment.

The BBB serves a critical role in regulating the molecular exchange between peripheral blood and the central nervous system. Maintaining proper functioning of the BBB is essential for brain health, although the full pathological relevance of its dysfunction is poorly understood [48,49]. However, research has linked BBB dysfunction to cognitive dysfunction, increased neuroinflammation [15], and neuropsychiatric disorders [16], suggesting that SR and damage induced by SR and other spaceflight stressors could alter emotion and impair cognitive function in astronauts in ways that could impact their ability to complete their mission. Thus, determining how singular and combination inflight stressors interact to modulate the immune system and BBB is crucial to fully understand the multiple pathways that could impact astronaut performance and health, including the alterations to the CNS structures and cell viability observed in this study.

## Data Availability

Experimental data available upon request.

## Supplementary Materials

The following supporting information can be downloaded at: https://www.mdpi.com/article/10.3390/life14050636/s1, Supplementary Figure S1: Stress Vulnerability Increased BBB Permeability and was Exacerbated by SR but Ameliorated by SI; Table S1: Summary of Results for Observed Differences in Astrocyte Morphology.
