# Supplementary material for: Alterations in Blood–Brain Barrier Integrity and Lateral Ventricle Differ in Rats Exposed to Space Radiation and Social Isolation
Source: Life (Basel). 2024 May 16;14(5):636. doi: 10.3390/life14050636 (PMC11122575; doi:10.3390/life14050636)
Supplement: Supplementary file 1 [file life-14-00636-s001.zip › life-2974161-supplementary.pdf]

life

Supplementary Materials

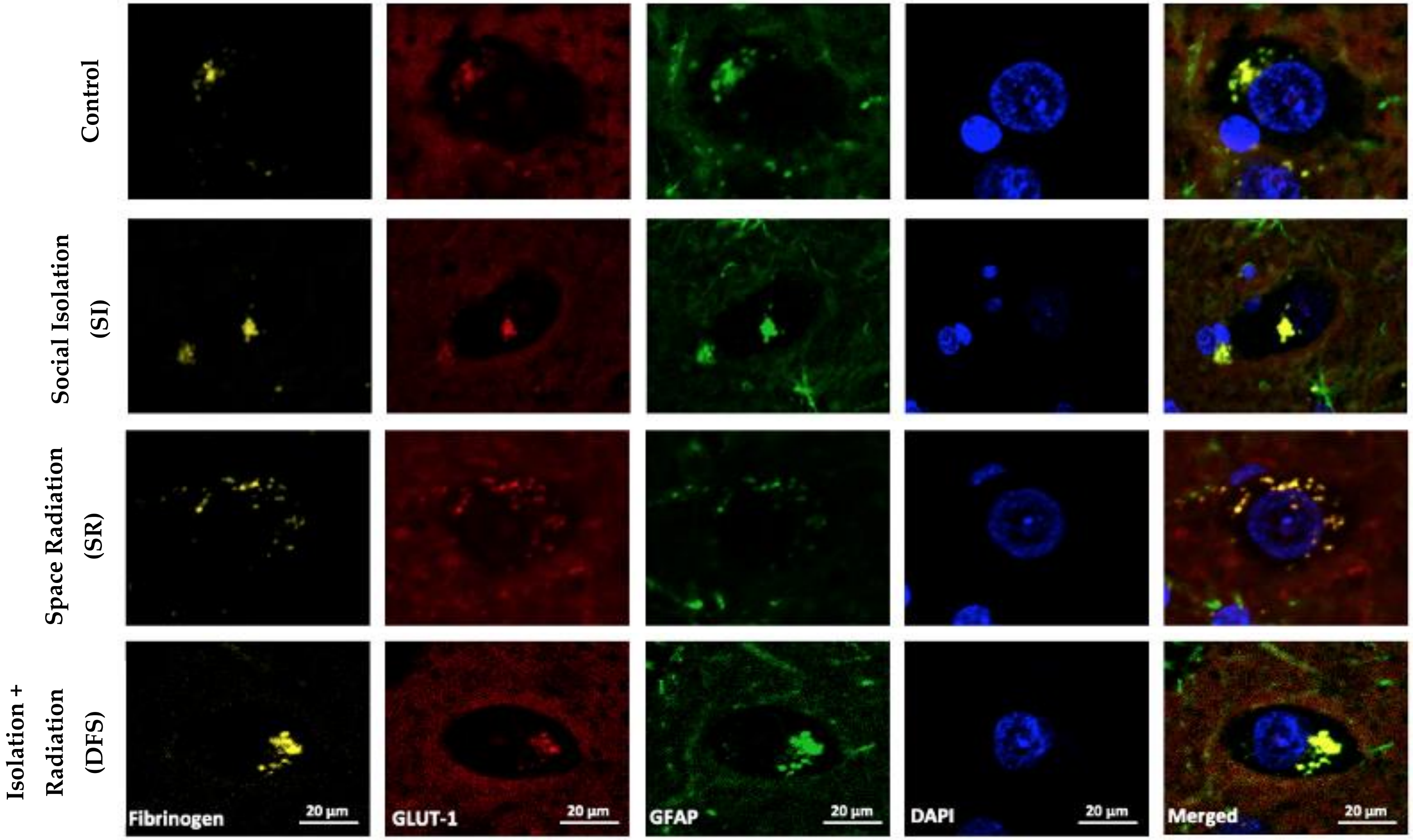

Supplementary Figure S1. Stress Vulnerability Increased BBB Permeability and was Exacerbated by SR but Ameliorated by SI. Quadruple-label immunofluorescent images of a single blood vessel stained with fibrinogen (yellow), Glut-1 (red), GFAP (green), and DAPI (blue) displaying vascular permeability of resilient (Res) and vulnerable (Vul) phenotypes in each treatment group. All images were acquired at 40x magnification and zoomed to focus on a single vessel. Scale bar= 20µm.

Table 1. Summary of Results for Observed Differences in Astrocyte Morphology. Table showing a summary of the overall results in astrocyte morphology between treatment groups.

	General Morphology	Average Projection Distance from Nucleus (μm)	Average Number of Projections per Astrocyte	Average Number of Branches per Projection
**Control**	Fibrous- 25%<br>Protoplasmic- 75%<br>Dead/No Projections- 0%	22.45 ± 1.67	3.75 ± 0.28	4.17 ± 0.53
**SI**	Fibrous- 70.59%<br>Protoplasmic- 29.41%<br>Dead/No Projections- 0%	17.77 ± 2.74	4.41 ± 0.44	2.94 ± 0.51
**SR**	Fibrous- 50%<br>Protoplasmic- 0%<br>Dead/No Projections- 50%	13.39 ± 1.02	1.94 ± 0.23	0.39 ± 0.12
**DFS**	Fibrous- 62.35%<br>Protoplasmic- 37.65%<br>Dead/No Projections- 0%	14.95 ± 1.55	2.24 ± 0.26	1.29 ± 0.48
